# Maximizing the impact of community outreach and engagement at US cancer centers

**DOI:** 10.1093/jncics/pkae053

**Published:** 2024-06-26

**Authors:** Shoba Ramanadhan, James Daly, Rebekka M Lee, Kamini Mallick, Samantha L Augenbraun, Karen M Emmons

**Affiliations:** Department of Social and Behavioral Sciences, Harvard T.H. Chan School of Public Health, Boston, MA, USA; Department of Social and Behavioral Sciences, Harvard T.H. Chan School of Public Health, Boston, MA, USA; Department of Social and Behavioral Sciences, Harvard T.H. Chan School of Public Health, Boston, MA, USA; Department of Social and Behavioral Sciences, Harvard T.H. Chan School of Public Health, Boston, MA, USA; NORC at the University of Chicago, Chicago, IL, USA; Department of Social and Behavioral Sciences, Harvard T.H. Chan School of Public Health, Boston, MA, USA

## Abstract

In 2016, the National Cancer Institute–designated cancer centers funding opportunity was expanded to require community outreach and engagement (COE), with explicit attention to cancer inequities, community engagement, and implementation science in the centers’ catchment areas. Resource limitations constrain these activities, and we conducted a qualitative study to understand what COE leaders see as critical needs and supports to increase impact. In the spring of 2021, we interviewed leaders from 56 of 64 cancer centers with COE programs and analyzed the data using a reflexive, thematic approach.

We identified 6 categories of needs: 1) centering community engagement among leadership and non-COE researchers, 2) increasing training on implementation science/practice, 3) improving integration into cross-center networks, 4) increasing funding for staffing and sustainment, 5) revising funder guidance and reporting, and 6) facilitating data utilization. COEs need long-term, systems-focused investments to engage communities, increase research translation, and advance health equity.

The National Cancer Institute (NCI)–designated cancer centers offer potential to meet the growing burden of cancer in the United States and globally as they conduct innovative research to advance prevention, diagnosis, and treatment of cancer (1). In the United States, the centers are vital for addressing substantial structural inequities related to cancer, from exposure to risk factors and access to preventive services to incidence and mortality, based on race, ethnicity, socioeconomic status, and rural/urban resident status (2). In 2016, the cancer centers’ funding opportunity was expanded to require community outreach and engagement (COE) (3). Although many cancer centers were already engaged locally, the expansion required cancer centers to focus on their catchment areas and conduct research to address cancer inequities, engage community members in research and agenda setting, conduct outreach and education, support representative clinical trial recruitment, and translate research into policy. They were also charged with influencing research and policy beyond the catchment areas (4).

Given these requirements, focusing on community-engaged research and implementation science is a natural fit. Community-engaged research offers opportunities to use collaborative efforts to address issues important to communities and produce knowledge and action to address health inequities (5). Implementation science offers the opportunity to support the integration of evidence-based interventions (EBIs) into routine health care and public health practice (6). This is critical given that more than half of the cancers occurring in the United States today could be prevented through the utilization of existing research evidence, and yet these solutions are not delivered sufficiently or equitably (7). By linking community engagement and implementation science, academic researchers and community partners can collaboratively leverage the best available research and practice/community expertise for cancer prevention and control. These linkages can improve health, reduce inequity, and create transformational change (8). Although COEs contain rich resources to address cancer inequities, they also face significant constraints that limit their ability to generate large-scale impact. We conducted a qualitative study to understand the ecosystem more thoroughly, including opportunities highlighted by COE leadership to increase impact.

At the time of this study (spring 2021), there were 71 NCI-designated cancer centers, 7 of which were basic laboratory centers and, therefore, excluded from our sample. Of the relevant 64 centers, we conducted 1-hour interviews with 63 COE directors and designees representing 56 centers from March to June 2021 (88% participation rate). The 8 centers that chose not to participate were spread across 6 states in varying regions of the country. This group included 6 comprehensive cancer centers. The study was deemed exempt by the Harvard University institutional review board, and informed consent was obtained from each participant. Interview topics covered 1) COE structure and priorities; 2) examples of how COE activities and interventions are identified, implemented, evaluated, and sustained, and factors influencing these efforts; and 3) needs and supports to enhance COEs’ ability to identify, implement, and sustain EBIs and conduct community outreach and engagement. The interview guide is available as Supplementary Methods (available online). The Consolidated Framework for Implementation Science guided interviews (9), which focused on implementation science but did not cover dissemination efforts. Interviews were recorded and professionally transcribed. We analyzed the data using a thematic analysis approach described by Braun and Clarke (10). We used a combination of deductive codes linked to the implementation science framework and inductive codes (eg, those linked to community engagement activities). We relied on team discussion to advance the analysis and used reflexive techniques to identify the boundaries and influences of our knowledge and experiences on the study. The author team has expertise in qualitative methods, community-engaged research, and implementation science, all from academic orientations. Several authors had experience working with cancer centers and COE programs and, in a few cases, existing relationships with interview participants.

This brief communication summarizes core needs and opportunities for support with community engagement and implementation science. As highlighted in Figure 1, we identified 6 main categories of needs:

**Figure 1.**
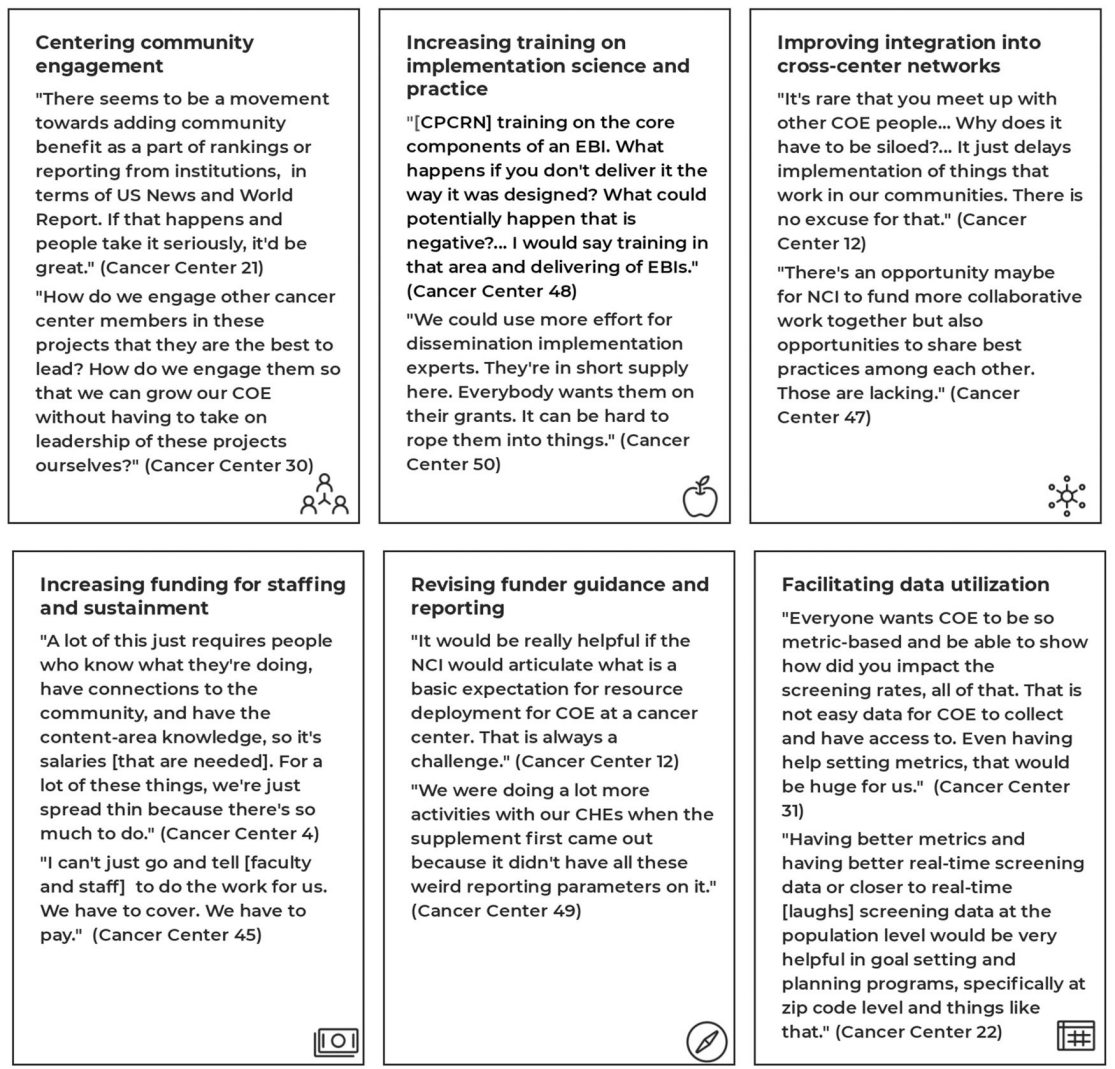
Core areas of need identified by cancer center community outreach and engagement (COE) leaders and staff, with exemplar quotes. CHE = community health educator; CPCRN = Cancer Prevention and Control Research Network; EBI = evidence-based intervention; NCI = National Cancer Institute.

1. **Centering community engagement**. Participants described a need to increase the value placed on community engagement in cancer centers, with many noting that center leadership did not appear to value the work of the COE. Participants also described needing support to prompt academics, particularly bench scientists, to engage with the COE and community-engaged research. One suggestion was to integrate COE efforts throughout the cancer center grant application. Another suggestion was to use targeted, internal funding for community-engaged research to promote interest among a broader range of researchers.
2. **Increasing training on implementation science and practice.** Participants highlighted the need to build a cohort of intermediate-level implementation scientists (instead of a few experts and many novices). Training requests also included implementation science, research basics, and program implementation for COE staff and partners. For example, participants suggested trainings on using an NCI database of EBIs (11) and the Putting Public Health Evidence into Action approach (12).
3. **Improving integration into cross-center networks**. Participants described interest in connecting across centers regarding partnership management (eg, paying community advisory board members), EBI use (eg, updated resources for finding them), and implementation strategies (eg, sharing a model for leveraging *promotoras*). Some participants noted that networking might reduce competition and increase collaboration among cancer centers.
4. **Increasing funding for staffing and sustainment**. Participants noted constraints on COE impact because of limited budgets. They described needing funds to conduct community-engaged research and implementation science, implement EBIs, evaluate programs, and support community engagement staff. They also emphasized needing resources to compensate staff fairly to increase retention and support partnership development and sustainment.
5. **Revising funder guidance and reporting**. Participants wanted to learn more from NCI about expectations related to infrastructure, partnerships, impact on cancer inequities, and responsiveness to community needs. They also sought guidance on allocating funds and staffing effort across COE priorities. Participants noted that the intense and highly structured COE reporting negatively impacted service delivery.
6. **Facilitating data utilization to address local cancer burden**. Related to data use, participants requested a variety of supports to address local needs. These included assistance with leveraging cancer registries (eg, SEER), creating and maintaining catchment area-level data sources, using electronic medical records for implementation evaluation, and engaging data scientists who could support impact evaluation.

As COE programs evolve, long-term, systems-focused investments can increase their impact. One set of changes relates to broadening the understanding of the value and utility of community-engaged research and community outreach and engagement. These complementary foci offer the opportunity to simultaneously engage in resource-intensive, engaged research projects that take time to yield results and more immediate action for outreach related to pressing concerns in the catchment area. It will be essential to build this support for community-engaged research and community outreach and engagement among cancer center leadership and a broader range of researchers. Strategic design of funding opportunities can promote such attention, and a recent study highlights the correlation between ratings for the COE component and the overall center rating among comprehensive cancer centers (13). Another set of opportunities lies in creating structures to increase researcher-community connections, which can integrate equity considerations into cancer center research more effectively, facilitate representative recruitment, and support disseminating results (14).

Long-term investments must also focus on building and sustaining capacity within COE programs for community-engaged research and implementation science. Given that capacity-building requires multiple, ongoing interactions (15), offerings could be integrated into routine cancer center activities (eg, through pilot grants and technical assistance). Existing resources (12,16-19) and newly created offerings can meet these needs. Similarly, long-term investments allow for the alignment of COE operations and values. For example, a commitment to advancing equity and collaborating through meaningful partnership must translate into appropriate compensation and resources for community partners and COE staff. Related, long-term investments support COE goals regarding meaningful catchment area engagement and translation of research into practice and policy in a way that discrete, short-term investments cannot. Finally, investments focusing on NCI’s central role with the cancer centers will support knowledge sharing and collaboration across COEs. These supports have been identified elsewhere as crucial to creating systems change and advancing health equity through cancer centers (20). Existing offerings, such as the annual convening of COEs, Catchment Area Data Conference, and the Consortium for Cancer Implementation Science, are excellent starting points. Another opportunity for coordination comes from connecting data and programming in cancer centers, particularly at the catchment-area level.

Our broad findings echo the summary report from a recent convening of COE programs. Key barriers included limited funding and resources (for staff, evaluation, and achieving the full COE mandate), support with data systems, support to address social determinants, and guidance on addressing policy translation (21). We present our findings within a set of limitations and strengths. First, the research question addressed in this analysis was not the primary focus of our interviews. However, the data were sufficiently rich for the narrow question, tightly specified and highly expert participant population, reliance on theory, and detailed responses we received (22). Second, participants may have felt social desirability pressures to respond in a particular manner, although we tried to limit this through the choice of interviewers and how questions were presented.

Overall, COEs at cancer centers represent a tremendous opportunity to bridge research-practice and community-academic gaps. By addressing the needs and supports identified above, COEs will be well positioned to achieve their potential and increase the translation of critical cancer prevention and control services to communities while advancing the core goals of advancing health equity.

## Supplementary Material

pkae053_Supplementary_Data

## Data Availability

The data underlying this article cannot be shared as that would compromise the privacy of the individuals who participated. Upon request, investigators can provide high-level summary data.
