# Supplementary material for: Maximizing the impact of community outreach and engagement at US cancer centers
Source: JNCI Cancer Spectr. 2024 Jun 26;8(4):pkae053. doi: 10.1093/jncics/pkae053 (PMC11245743; doi:10.1093/jncics/pkae053)
Supplement: pkae053_Supplementary_Data [file pkae053_supplementary_data.pdf]

Supplementary Materials

Supplementary Methods

COE LEADER INTERVIEW GUIDE

PLACEHOLDER FOR CONSENT PROCEDURES

COE BACKGROUND STRUCTURE AND PRIORITIES

First, I'd like to talk to you about your organization and your cancer-related priorities.

1. Can you briefly describe the overall structure of your center's Community Outreach and Engagement program; who is involved in your COE activities?

POTENTIAL PROBES:

- Who in your organization is responsible for managing or maintaining this/these project(s)?
- Who assists in COE activities (position/title)?
- Who interacts directly with community partners (position/title)?
- Who are the core community partners you work with on these project(s)?
- Does your COE include a Community Advisory Board (CAB)? **If yes,** can you describe the CAB structure?
- What is your role or involvement in COE program activities?

2. Briefly, what has your center identified as the top cancer-related priorities and needs in your catchment area?

POTENTIAL PROBES:

- What cancer and cancer risk factors are top priorities?
- Are there any groups that have been identified as priority populations?

PROGRAM IDENTIFICATION, IMPLEMENTATION, EVALUATION, AND SUSTAINMENT

Next, I want to talk about how your center identifies and implements relevant projects. For this and all topics that we will discuss, there are no right or wrong answers -- every center will have different projects, priorities, and approaches based on their unique needs. We are interested in learning more about the **variety** of approaches across centers, and what works in your center and community.

Can you briefly describe an example of community outreach and engagement activities at your center?

FOR THE ACTIVITY/INTERVENTION – ASK QUESTIONS AS NEEDED,
IF NOT ALREADY ADDRESSED IN DESCRIPTION:

3. How did you go about identifying and selecting < ACTIVITY/INTERVENTION >?

POTENTIAL PROBES:

- How do you consider the scientific literature (e.g., published studies, resources including the NCI Evidence-Based Cancer Control Programs [RTIPS]) when selecting this intervention/approach?
- What was the role of the Cancer Center's faculty in selecting these activities/interventions?

  - Are there any faculty with specific expertise that you typically consult when selecting interventions? What type of expertise do they have (e.g., subject matter, cancer type, dissemination/implementation – those who study how to put programs in place)?
  - How do you solicit this input or feedback from faculty?
- What was the role of the Community in selecting these activities/interventions?
  - How do you solicit community input or feedback (e.g., community advisory boards)?
- To what extent do you consider:
  - Characteristics of the intervention/approach
  - Organizational infrastructure (e.g., available resources, size, physical layout, internal policies, leadership)
  - Characteristics of individuals within the organization (e.g., role and experience of those involved)
  - Influences outside of the organization (e.g., Local, state, or national policies; community characteristics; income, race, ethnicity of population served).

4. Where are you at in the process of implementing < ACTIVITY/INTERVENTION >, and how has it gone? (early in the process, well under way, done; didn't go well, went as planned, had/didn't have expected outcomes)

5. What factors influenced your efforts to implement this intervention?

POTENTIAL PROBES:

- Characteristics of the intervention/approach
- Organizational infrastructure (e.g., available resources, size, physical layout, internal policies, leadership)
- Characteristics of individuals within the organization (e.g., role and experience of those involved)
- Influences outside of the organization (e.g., Local, state, or national policies; community characteristics; income, race, ethnicity of population served).

6. Can you describe some of the approaches you and your team have used to identify potential barriers and facilitators to implementing this project?

POTENTIAL PROBES:

- What was the role of the Cancer Center's faculty in identifying barriers or facilitators; in developing approaches to address potential barriers?
  - Are there any faculty with specific expertise that you typically consult when developing approaches to address potential barriers? What type of expertise do they have (e.g., subject matter, cancer type, dissemination/implementation)?
- What was the role of the Community in identifying potential barriers or facilitators to this project?
  - How do you solicit community input or feedback (e.g., community advisory boards)?
- How does this type of information – identifying potential barriers or facilitators – impact your intervention approach?
  - How do you address potential barriers?
  - How do you capitalize on implementation facilitators?

7. What type of information do you gather to assess the effectiveness of < ACTIVITY/INTERVENTION > and who is involved in that process?

POTENTIAL PROBES:

- What was the role of the Cancer Center's faculty in assessing the success of this activity?
- What was the role of your community partner in assessing the success of this activity?

8. What approaches has your center used to help sustain < ACTIVITY/INTERVENTION > that address your catchment area priorities?

POTENTIAL PROBES:

- Enhancing/supporting outer context (e.g., leadership, policies, funding, networks, client characteristics, advocacy)
- Enhancing/supporting inner context (e.g., leadership, implementation expertise, organizational characteristics, fidelity monitoring/support)
- Enhancing/supporting bridging characteristics (e.g., community partnerships)
- Enhancing/supporting innovation adaptability

[FOR EACH ACTVITY/INTERVENTION DESCRIBED, Interviewer should summarize which of the descriptions below they believe best describes the process used and ask for confirmation from respondent as to whether that best reflects the process.]

- Shared information related to cancer risk factors in your catchment area, that your community partners could act upon? (Inform)
- Sought out input or feedback from community organizations? (Consult)
- Worked with your community to gather information and understand issues or problems related to cancer-related priorities and needs in your catchment area? (Involve)
- Developed partnerships within your catchment area to seek advice and input on cancer-related priorities? (Collaborate)
- Worked with community partners and empowered them to make decisions about cancer-related priorities and approaches in your catchment area? (Empower)

SECOND ACTIVITY/INTERVENTION

Thank you for walking me through that project. Is there a second example of a community outreach and engagement project that you can walk me through?

FOR SECOND ACTIVITY/INTERVENTION – REPEAT QUESTIONS/PROBES 3-8 AS NEEDED,
IF NOT ALREADY ADDRESSED IN DESCRIPTION:

NEEDS AND SUPPORTS

8. Finally, what type of support does your COE need to enhance your current community outreach and engagement activities in the future?

POTENTIAL PROBES:

- Outer context (e.g., leadership, policies, funding, networks, client characteristics, advocacy)
- Inner context (e.g., leadership, implementation expertise, organizational characteristics, fidelity monitoring/support)
- Bridging characteristics (e.g., community partnerships)
- Innovation adaptability

PLACEHOLDER FOR WRAP UP AND NEXT STEPS
